# Investigating the relation between positive affective responses and exercise instigation habits in an affect-based intervention for exercise trainers: A longitudinal field study

**DOI:** 10.3389/fpsyg.2022.994177

**Published:** 2022-09-23

**Authors:** Susanne Weyland, Julian Fritsch, Katharina Feil, Darko Jekauc

**Affiliations:** Department of Health Education and Sports Psychology, Institute of Sports and Sports Science, Karlsruhe Institute of Technology, Karlsruhe, Germany

**Keywords:** habit formation, instigation habit, affective response, affective attitude, intervention development

## Abstract

The present study contains an affect-based intervention intended to support exercise trainers in positively influencing their course participants’ affective responses to their exercise courses. We argue that positive affective responses are associated with habit formation, thereby being a promising approach for avoiding high drop-out rates in exercise courses. First, the present study aimed to investigate whether the intervention for exercise trainers could increase (a) affective attitudes, and (b) exercise instigation habit strength, and influence the development of (c) weekly measured affective responses and (d) automaticity among adult participants of exercise courses. Second, it examined the relationship between the development of affective responses and exercise instigation habit strength. Ten exercise trainers of weekly sports and exercise courses at a German university received either an affect-based intervention or a control intervention. 132 of their course participants answered the Self-Report Habit Index (SRHI; the automaticity sub-scale SRBAI was also analyzed) for exercise instigation habit strength and items to measure affective attitude in the initial and final assessment. Moreover, they were assessed for a duration of 10 weeks during which, each time after attending the course, they reported their affective response to exercise as well as their automaticity in arriving at the decision to exercise. In the repeated measures ANOVA, there was a significant main effect of time for exercise instigation habit strength. Overall, habit strength was higher in the final than in the initial assessment. However, there were no significant differences between the two conditions in all study variables. In the latent growth curve model, the trajectory of the latent growth curve of valence was a significant predictor of the final exercise instigation habit strength. While the applied affect-based intervention was not successful in enhancing positive affective responses to exercise, the results indicate that positive affective responses may contribute to strengthening exercise instigation habits. Future studies should examine the effectiveness of interventions in long-term study designs.

## Introduction

Physical activity (PA) is good for you: more precisely, preventive health benefits, e.g., a 20–30% risk reduction for about 25 chronical diseases, derive from regular PA (Powell et al., 2011; Rhodes et al., 2017). Most adults are aware of exercise recommendations, with 68% of one study’s American respondents correctly identifying specific PA guidelines (Morrow et al., 2004). Accordingly, in a recent study following German voluntary university sports and exercise courses for 13 weeks, the means of the weekly intention to re-attend the course next time were constantly at around 9, on a scale from 1 to 10, where 10 corresponded to the strongest intention (Finne et al., 2019). However, it turned out that after the courses’ fourth week, not even half of the initial participants were present. Thus, the research interest on what constructs might bridge this intention-behavior gap in the long run and increase exercise course re-attendance rates arises (for a meta-analysis quantifying the intention-behavior gap in a PA context, see Rhodes and de Bruijn, 2013). The model of physical activity adoption and maintenance (PAAM model, Strobach et al., 2020), which is a dual-process model, assumes that particularly for behavior maintenance it is important to also take affective and automatic processes into account.

The affective response to PA is an affective state, which refers to how an individual feels in response to acute PA (Stevens et al., 2020). The hedonic principle assumes that people seek pleasure and avoid displeasure (e.g., Williams, 2008), which is supported by numerous empirical findings that a positive affective response during PA is positively related to future PA (Rhodes and Kates, 2015; Williams et al., 2016). According to the Affective-Reflective Theory of physical inactivity and exercise (ART, Brand and Ekkekakis, 2018), which is a dual-process model, affective responses can influence exercise behavior in two ways. First, through an automatic affective valuation, the impact can be direct, leading to an action impulse (type-1 process). Second, affective responses can have an indirect impact in that they influence deliberative reasoning, resulting in action plans (type-2 process). In their recent narrative review, Stevens et al. (2020) refer to the cognitive processing of experienced affective responses as “affect processing” (see also Williams and Evans, 2014). Accordingly, unlike affective responses, affect processing does not represent acute affect per se and can be invoked and measured outside the very situation of the target behavior. An example for affect processing is the affective attitude. Concerning the relationship between affective responses per se and affective attitudes, Stevens et al. (2020) argue that an affective attitude is theoretically formed after remembering the actual affective response first and then anticipating a future affective response. Accordingly, the affective attitude is per definition based on both probability and individual importance of affective outcomes. A meta-analysis has shown that affective judgements, to which, according to Stevens et al. (2020), the affective attitude belongs, are positively related to PA (overall *r* = 0.42) in adult samples (Rhodes et al., 2009). Therefore, in this study, the focus lies on implicit affective responses and explicit affective attitudes, which are influenced by affective responses.

There are several approaches on how to manipulate affect-based constructs through interventions (for an overview see Conner et al., 2020; Chen et al., 2021). With regards to the potential content of interventions targeting affective responses, the qualitative study of Wienke and Jekauc (2016) identified four facilitators of positive affective responses in exercise, namely perceived competence, perceived social interaction, novelty experience, and perceived physical exertion. These four facilitators were also found to be related to positive affective responses in other studies. Leisterer and Jekauc (2019) showed in two separate studies that positive competence-based performance feedback and the experience of partner exercises were positively related to positive affective responses. Further, perceived variety was found to be positively related to indices of exercise-related well-being (i.e., positive affect; Sylvester et al., 2016). Also, several studies exist that link self-selected exercise intensity to positive affective responses (for a review see Ekkekakis et al., 2011). A resource-saving approach that seems feasible in a university sports context could be to educate exercise trainers on how to elicit positive affective responses in course participants based on the identified facilitators. One intervention conducted by Jekauc (2015) educated exercise trainers on promoting positive affective responses in their course participants based on, amongst other things, autonomy (e.g., self-selected intensity and exercises), competence (e.g., giving positive feedback), and relatedness (e.g., choosing group exercises over single exercises). While enjoyment as an indicator of positive affective responses decreased in the control group during the first 4 weeks, it increased in the intervention group. The results in two studies by Strauch and colleagues (Strauch et al., 2018, 2019) suggest that an exercise trainer’s personal appearance and interactions with course participants have a major impact on course participants’ affective responses. They found that the ability to manage one’s own emotional expression is part of the coach competences that together lead to the generation of positive affective responses in participants of sport and exercise programs. This might be explained by the phenomenon of emotional contagion (Hatfield et al., 2014). These results reinforce the idea of a trainer-focused intervention, and also suggest a concrete way to manipulate affective responses, namely through the exercise trainer’s emotional expressions themselves and not just through their behavior.

The other implicit construct on which this study focuses is habit. Habit is defined by Gardner and Lally (2018, p. 207) as “a process whereby encountering a cue triggers an impulse to perform an action that has, through learning, become a learned response to the cue.” Orbell and Verplanken (2020) outline three basic components of habit, namely the repetition of a behavior as response to a consistent cue, the development of mental cue-behavior associations, and the resulting cue-dependent automaticity. Automaticity means performing a behavior, for example, without thinking and without having to consciously remember it (Gardner et al., 2012). The more global construct of habit measured by the Self-Report Habit Index (SRHI; Verplanken and Orbell, 2003) includes frequency and relevance to one’s self-identity in addition to automaticity. Automaticity can be understood as a key characteristic of habitual behavior and refers to the moment of the specific behavior. The frequency of a behavior is rather to be seen as a determinant of habit and self-identity might be a consequence (see also the reflection on the SRHI by Sniehotta and Presseau, 2012), which gives the construct habit measured by the SRHI a different degree of specification. If a cue triggers the initiation of a behavior out of other alternative behavioral possibilities, it is called instigation habit, whereas triggering sub-behaviors within a given behavioral sequence is called execution habit (Gardner et al., 2020b). One study concluded that building instigation rather than execution habits is related to changes in future exercise frequency (Phillips and Gardner, 2016). Positive correlations between habit strength and PA were also revealed in a current systematic review, which included longitudinal studies (Feil et al., 2021). Given the assumption that a behavior is automatically triggered by a cue, habit might contribute to exercise maintenance in that it bridges fluctuations in motivation and helps maintain the behavior even when rewards are removed (Neal et al., 2011; Rebar et al., 2019). Since cue-behavior associations characterize habits, habit formation theoretically requires cue-consistent repetitions of a behavior (Gardner et al., 2020a). According to Gardner and Lally (2018), variables influencing the habit formation process can at first support individuals in building the intention to perform the behavior. Also, they can help initiating and maintaining the behavior under constant conditions. Moreover, they can act as moderators of the repetition-habit relation and thereby influence the developing mental cue-behavior association. The latter is all the more important since research shows that habit strength forms at different rates, despite an equal number of repetitions (Lally et al., 2010).

Linking habit to affect, a behavior might be repeated more often if accompanied by positive affect. This can be theoretically justified by the “law of effect,” according to which responses to a situation that entail satisfaction are more likely to occur again in that same situation (Thorndike, 1911). Also, it can be argued that the relation between the mere repetition of a behavior and habit strength can be moderated by rewards (de Wit and Dickinson, 2009; Lally and Gardner, 2013). Accordingly, there is research suggesting that the cue-behavior association characteristic of habits might be strengthened through repetition, and each repetition might contribute more to habit formation when perceived as a reward (Wiedemann et al., 2014; Schnauber-Stockmann and Naab, 2019). Wiedemann et al. (2014) view affective constructs, namely satisfaction or pleasure, as examples for intrinsic rewards. A behavior that was accompanied by the experience of a positive affective response may be triggered impulsively in the next moment of decision making because the automatic affective valuation is positively valenced and no restraining forces counteract it, as can be argued with the ART (Brand and Ekkekakis, 2018). This type-1 process is fast and automatic, actually reflecting the idea of instigation habits, where a behavior is instigated before the individual realizes it, i.e., before type-2 processes have followed. Radel et al. (2017) found that self-determined motivations (e.g., intrinsic motivation) were stronger related to behavioral automaticity than non-self-determined motivations (e.g., extrinsic motivation) and that self-determination moderated the relationship between repetition of a behavior and behavioral automaticity. The authors explain their findings with the affective states associated with the different motivations, suggesting that positive affective states might be related to intrinsic motivation. They argue that positive affective states lead the individual to rely on automatic processes which would not be equally the case had negative affective states been associated. Further, although theoretically unexpected, motivational or rewarding variables were found to directly predict habit formation of health behaviors independently of repetition: Gardner and Lally (2013) found this direct effect for self-determined motivation, Judah et al. (2013) found it for favorable attitudes, and Weyland et al. (2020) for affective responses. Regarding affective attitude, one cross-sectional study showed a significant positive correlation with exercise habit strength (de Bruijn and Rhodes, 2011). Referring to data from new gym members, Kaushal et al. (2018) found that affective judgement, together with behavioral regulation and preparatory habit (i.e., automatically preparing for exercise), collectively explained mediation between condition (an educative workshop that underlined the importance of self-regulative action planning, consistent use of cues, and rewards vs. control) and change in self-reported PA. Another variable from the affect processing domain is enjoyment, according to Stevens et al. (2020), and enjoyment was also shown in one study to positively predict exercise habit (Teixeira et al., 2022).

Considering the relationship between affective responses, habit, and thus behavior, affect-based interventions might not only lead to more positive affective responses to exercise and consequently to more positive affective attitudes, but also reinforce habit formation. Therefore, the primary objective of the present longitudinal study was to examine the effectiveness of a trainer-focused affect-based intervention to promote affective attitudes and habit strength as well as to influence the development of affective responses and automaticity among adult exercise course participants. The secondary objective was to examine whether the development of affective responses is related to the development of habit strength. In accordance with the primary objective, we hypothesized that a trainer-focused intervention, focusing on the induction of positive affective responses in exercise course participants (affect condition) compared to a trainer-focused intervention with a purely physiological content (control condition), would result (1a) in more positive affective attitudes towards exercise, and (1b) in higher exercise instigation habit strength. Further, we hypothesized that the intervention would influence the development of (1c) positive affective responses to exercise, and of (1d) automaticity among course participants. Moreover, regarding the secondary objective of the study, we hypothesized that the development of positive affective responses to exercise is positively related (2) to the development of habit strength in the overall adult sample.

## Materials and methods

### Participants

Over the course of 10 weeks, data were collected from weekly sports and exercise courses offered by the department of university sports to all students and employees of one German university during the winter semester. The 10 selected sports and exercise courses covered basketball, yoga, badminton, table tennis, field hockey, and volleyball. Participants in the study from whom the questionnaire data were collected were participants of these courses who volunteered to participate in the study and provided written informed consent. Eligible participants of the exercise trainers’ courses had to be at least 18 years old, feel physically healthy, and understand German. From those present in the first week of the study, 135 students and employees agreed to participate in the study (100 male, 33 female, 2 missing; mean age 22.30 years; 129 students, 4 employees, 2 missing). Sixty-six were in the affect condition, and 69 in the control condition. The study was approved by the university’s data security commissioner and ethics committee.

### Procedures

The exercise trainers were recruited via email after having consulted their head of university sports of their German university. This email informed them that the purpose of the study was to test the effectiveness of trainer-focused interventions in reducing drop-out rates in university sports and exercise courses and that participation would be voluntary. Participation in the intervention, for which individual appointments were made with the exercise trainers, did not obligate them to also participate in the study. Eligible exercise trainers had to lead courses that (a) were accessible to students and staff, i.e., without special restrictions (e.g., high costs, such as for golf, could influence participation), (b) were not related to official competitions (as competitions were seen as a type of participation obligation), (c) were led by the same exercise trainer every week, and (d) were theoretically eligible for the implementation of the intervention content (e. g., diving courses were excluded). Given the quasi-experimental design of the study, another prerequisite for participation in the study was that two different exercise trainers instructed the respective courses where two similar courses of the same kind of exercise were offered.

Of the 72 exercise trainers contacted, we received a response from 14 who stated that they were interested in the workshop. For matched randomization, a coin toss, whilst considering “kind of exercise” and “course level” (from beginners to advanced), was used to assign the exercise trainers’ courses to affect or control condition. For example, the two different advanced courses and the two different table tennis courses were matched, respectively. In total, 10 exercise trainers met the inclusion criteria and were randomly assigned to either affect (*N* = 5) or control condition (*N* = 5). Exercise trainers of these courses were informed and gave their consent, but were not asked to complete any questionnaires. During the first weeks of the winter semester 2019, a one-hour workshop for the affect condition was conducted by the lead investigator, at the same time, a workshop for the control condition was conducted by two sport science students. Exercise trainers were told which condition they were in, but they were blind to the other condition. Since organizational matters are settled in the first sessions of the university exercise courses, and many interested individuals just drop by to have a look, data collection began in the fourth week of the semester. In the first week of data collection, individuals who agreed to participate completed the first questionnaire (initial assessment) and the first of the weekly short questionnaires. During the study, all courses were attended by the study team on a weekly basis to collect data of all attending study participants. In the tenth week of data collection, the final questionnaire (final assessment) was additionally handed out after the course to all available study participants and, in case a person did not attend the course in the tenth week, also 1 week later.

#### Affect condition

The affect-based intervention for exercise trainers in the affect condition is based on the findings from Wienke and Jekauc (2016), namely on the four facilitators of positive affective responses in exercise perceived competence, perceived social interaction, novelty experience, and perceived physical exertion. It comprised a one-hour workshop, a summarizing laminated diagram (Supplementary Figure A), an information booklet (Appendix A) and a printed version of a manual for one get-to-know game. In the workshop, the four facilitators were explained, and techniques on how to implement the facilitators were discussed with the exercise trainers. They were not just taught, but it was also listened to their needs in order to take advantage of the benefits of tailored interventions suggested by the literature, such as a greater personal connection to the intervention material (see for example Kreuter et al., 1999; Kroeze et al., 2006). For example, when educating the exercise trainers on the facilitator social interaction, possible techniques were discussed and a game instruction was handed out as an example of a social support technique. In addition to the four facilitators, we also emphasised that the coaches should try to become aware of their own affective states and emotional expressions. This is based on the finding that the ability to manage one’s own emotional expressions leads to the generation of positive affective responses in participants of sport and exercise programs (Strauch et al., 2018, 2019).

#### Control condition

The intervention for exercise trainers in the control condition was designed to provide a benefit for the participating exercise trainers as well while at the same time not containing any relevant aspects of the affect-based intervention. It comprised a one-hour workshop and an information booklet. The workshop contained topics from current research in training science, such as aspects of warm up and cool down, motor skills as well as variations in coordination training.

### Measures

For an overview of measures and assessment times see Supplementary Figure B.

#### Initial assessment (week 1)

*Habit strength (exercise instigation)/Automaticity.* We measured exercise instigation habit strength in week 1 with a slightly adapted item stem of the German version of the Self-Report Habit Index (SRHI; Verplanken and Orbell, 2003), which was validated in its original version by Thurn et al. (2014). The wording of the item stem was, literally translated, “Going to an exercise course is something…,” which was inspired by the English item stem by Phillips and Gardner (2016), who reported face-validity in that the stem rather taps the decision than the execution. Since the exercise courses in this study were just about to start, we did not expect the participants to have already formed an instigation habit and, therefore, asked about exercise courses in general. The item stem was to be answered with 12 responses on a 5-point Likert scale (“strongly disagree” to “strongly agree”). Habit strength was calculated as the mean of the 12 items, whereby a high mean indicated high habit strength. Cronbach’s alpha was *α* = 0.89.

Additionally, we calculated the Self-Report Behavioural Automaticity Index (SRBAI). The SRBAI is a four-item automaticity sub-scale of the SRHI (Verplanken and Orbell, 2003), which had previously been validated (Gardner et al., 2012). A high mean of these four items indicated high automaticity. Here, Cronbach’s alpha was *α* = 0.86.

*Affective attitude.* We assessed the affective attitude towards exercising similar to de Bruijn et al. (2014). The item stem was, “Exercising for me is…,” and was answered on three 7-point bipolar adjectival scales, ranging from “very unpleasant” to “very pleasant,” from “very unenjoyable” to “very enjoyable,” and “very stressful” to “very relaxing.” A high mean of the three items indicated a more positive affective attitude towards exercising. Cronbach’s alpha was *α* = 0.78.

#### Weekly short assessment (weeks 1–10)

*Automaticity.* In order to keep the weekly questionnaire short for feasibility, we assessed automaticity as a characteristic of habit strength with one item comprising two item wordings from the Self-Report Behavioural Automaticity Index (SRBAI; Gardner et al., 2012). This one item was: “I went to this exercise course today automatically, without thinking.”

*Affective valence.* To measure affective responses to exercise, we applied a slightly adopted version of the widely used and, according to Backhouse et al. (2007), satisfactorily validated single-item bipolar Feeling Scale (Hardy and Rejeski, 1989) to answer the question, “How do you feel right now?”

While, according to the original versions, SRHI-items should be answered on at least a 5-point Likert scale and the Feeling Scale is an 11-point bipolar scale, that ranges from-5 (“very bad”) to 5 (“very good”), similar to Weyland et al. (2020), we modified the response format to bipolar 10-point scales for both weekly measures (ranging from “strongly disagree” to “strongly agree” for the automaticity item and from “extremely bad” to “extremely good” for the Feeling Scale) in order to better align with previous research.

#### Final assessment (final week)

*Habit strength/Automaticity and affective attitude.* We assessed habit strength/automaticity and affective attitude just like in the initial assessment (week 1). Cronbach’s alpha was *α* = 0.88 for exercise instigation habit strength, *α* = 0.85 for the automaticity sub-scale, and *α* = 0.74 for affective attitude.

### Statistical analyses

According to a meta-analysis by Chen et al. (2020), the expected effect size of a PA intervention targeting affective variables on affective variables is *r* = 0.26. A priori power analysis using G Power 3.1.9.7 indicated that for two repeated measures in a repeated measures ANOVA (within-and between-measures interaction), *N* = 72 participants was sufficient to detect an effect size of 0.20, using a rather conservative estimate, with *p* < 0.05 and power adjusted to 0.80. As a preliminary analysis, we screened the data for missing values and checked their patterns with Little’s MCAR test (*χ*^2^; Little, 1988) to decide on how to deal with missing data (Jekauc et al., 2012). Data were missing completely at random (see Appendix B for the results of the analyses of missing values). Thus, we decided that it was appropriate to use the expectation–maximization algorithm for data imputation to avoid list wise deletion in the case of analyses of variance (Dempster et al., 1977) and to use full-information maximum-likelihood estimation in the case of latent growth curve modeling (Arbuckle, 1996; Jekauc et al., 2012). Two-tailed independent sample t-tests and chi-square tests were conducted to check differences between the two conditions (affect versus control) regarding all variables assessed in the initial assessment at week 1 (gender, age, student status, habit strength, and affective attitude). For the analysis of the primary objective, examining the effects of the affect-based intervention on affective attitudes (Hypothesis 1a) and exercise instigation habit (Hypothesis 1b), two 2 (condition: affect versus control) × 2 (time: initial versus final assessment) repeated measures ANOVAs were conducted, using IBM SPSS 25 (Armonk, NY). Partial eta square was calculated to examine effect sizes. The threshold for significance was 0.05 for all analyses. Analyses of habit strength were conducted primarily with SRHI scores (automaticity, frequency, self-identity). Likewise, the analyses were calculated with the SRBAI values (automaticity only) to see if they differed.

For the analysis of the primary objective, to analyze the effects of the affect-based intervention on the development of the weekly measured variables affective responses to exercise (Hypothesis 1c) and automaticity (Hypothesis 1d), we applied latent growth curve modeling with IBM SPSS Amos 26 (Arbuckle, 2019), using a structural equation modeling framework. Moreover, for the analysis of the secondary objective, examining the relationship between the development of weekly measured affective responses and the development of habit strength (Hypothesis 2), we also applied latent growth curve modeling. To determine the overall goodness of fit of the models, chi-square statistics (χ^2^) are reported with *p*-values larger than 0.05, indicating that the model is fitting (Barrett, 2007). However, to avoid problems due to the small sample size, we additionally applied the comparative fit index (CFI) to evaluate the proposed model based on its relative improvement to the initial model – with values less than 0.90 indicating that the proposed model could still be improved considerably, values between 0.90 and 0.95 indicating acceptable fit, and values greater than 0.95 indicating good fit (Bentler and Bonett, 1980; Hu and Bentler, 1999; Hooper et al., 2008). Further, the root mean square error of approximation (RMSEA) was applied. According to Browne and Cudeck (1993), an RMSEA value of 0.05 or less is considered good and a value between 0.05 and 0.08 is considered acceptable. Moreover, the lower limit of the confidence interval of the RMSEA should be around 0 and the upper limit less than 0.08 in order to indicate a good fit (Hooper et al., 2008).

We first calculated separate models for valence and automaticity, respectively, to assess the development of both variables. For automaticity, additionally to a linear slope, we added a quadratic slope (fixing the paths at 0, 1, 4, 9 and so on) since there is evidence for a non-linear growth of habit strength (Lally et al., 2010; Schnauber-Stockmann and Naab, 2019). Regarding the model with valence only, chi-square statistics indicated that there was no significant difference between the postulated model and the data [*χ^2^* = 61.80, *df* = 50, *p* = 0.122; CFI = 0.89, RMSEA = 0.04 (90% CI 0.00–0.07)]. The slope mean of valence was −0.03 (*SE* = 0.02, *p* = 0.062), indicating that the linear trend of valence did not differ from zero. There were no inter-individual differences in the linear trend as indicated by a non-significant slope variance of valence (*σ^2^* < 0.01, *SE* = 0.01, *p* = 0.446). The correlation between intercept and slope of valence was not significant (*r* = 0.02, *p* = 0.392). The model with only automaticity and two latent slope factors revealed acceptable fit indices [CFI = 0.92, RMSEA = 0.07 (90% CI 0.04–0.10)], with *χ^2^* = 75.12, *df* = 46, *p* = 0.004. The non-significant slope means of automaticity were 0.13 (*SE* = 0.10, *p* = 0.221) for the linear slope and − 0.01 (*SE* = 0.01, *p* = 0.371) for the quadratic slope, respectively, indicating only very marginal changes over time. There were inter-individual differences in growth patterns, given the significant slope variances (linear: *σ^2^* = 0.45, *SE* = 0.18, *p* = <0.05; quadratic: *σ^2^* = 0.01, *SE* < 0.01, *p* = <0.05). The correlation between intercept and slopes of automaticity was not significant (linear: *r* = −0.40, *p* = 0.268; quadratic: *r* = 0.03, *p* = 0.475).

Given the non-significant correlations between intercept and slope within both separate models, we removed the covariance path between intercept and slope for both valence and automaticity in all following models. We calculated separate models for assessing the effects of the intervention on the development of affective responses (Hypothesis 1c) and on the development of automaticity (Hypothesis 1d). One of which examined the effect of the intervention on intercept and slope of valence and the other of which examined the effect of the intervention on intercept and slope of automaticity. Further, we examined whether the intercept and/or slope of valence would have an effect on final habit strength as well as/or on the change of habit strength (Hypothesis 2).

### Criteria for investigating habit formation

We were taking into account the essential criteria that must be met in order to investigate habit formation published by Gardner et al. (2022). First, we were focussing on the strengthening of the association between a cue, which might have been the date of the exercise course, and a behavior, i.e., instigating to go exercising. Second, it was reasonable to expect that this association would increase during the courses. The courses in this study had just started after the semester break, so even if someone was no first-year student and had already taken a similar course, there was a period of time in between when the behavior was not performed cue-congruently in the stable university setting. In addition, drop-out rates in such courses are high (Finne et al., 2019), so at least not all participants are at a stage in the habit formation curve where there is no meaningful growth. Further, the affect-based intervention aimed to result in some kind of “intrinsic reward” that would strengthen habit formation. Third, operationalizing habit as both a broad construct consisting of frequency, self-identity, and automaticity, as well as as automaticity alone, we were not simply inferring habit from the frequency. Fourth, by applying a structural equation modeling framework, we considered the continuity, non-linearity, and individual growth patterns of habit.

## Results

### Descriptive analyses

From the total sample size of 135 students and employees, two persons were excluded from the analyses since only data from the initial assessment in week 1 (no weekly or final assessment) was available, and one person was excluded since no data from the initial assessment was available. Thus, data from a total of 132 individuals were included in the analyses. According to an intention-to-treat analysis, no additional subjects were excluded prior to the analyses.

Thirteen participants (9.8%) attended all 10 courses, 61 participants (46.2%) attended the last course (12 participants filled in the final questionnaire 1 week later, therefore *N* = 73 final questionnaires were available). For the overall sample of 132 participants, mean participation rate was 6.55 times (*SD* = 2.63, range 1–10). Mean age was 22.31 years (*SD* = 2.25, range 18–29), 32 participants (24.2%) were female, 127 participants (96.2%) were students. At week 1, there were no statistically significant differences between the two conditions on sociodemographic data, habit strength (exercise instigation), and affective attitude (Table 1). See Supplementary Table A for correlations of all study variables.

**Table 1:** Group comparison of the initial assessment (week 1).

	Initial assessment (*N* = 132)				
	Affect (*n* = 65)	Control (*n* = 67)			
	*%*	*%*	*χ^2^*	*df*	*p*
Gender (female)	27.7	20.9	0.93	1	0.336
Student Status (yes)	95.4	97.0	<0.01	1	1.000
	*M (SD)*	*M (SD)*	*t*	*df*	*p*
Age	22.68 (2.22)	21.96 (2.24)	1.86	128	0.065
SRHI	3.34 (0.89)	3.36 (0.77)	−0.15	130	0.884
SRBAI	3.23 (1.10)	3.12 (0.97)	0.57	130	0.567
Affective attitude	5.71 (1.07)	5.62 (1.13)	0.47	130	0.637

The variables were measured after the participants had attended the course in the first week of the study. Habit strength was measured on a 5-point Likert scale; affective attitude on a 7-point scale. Statistics reported are percentages for the categorical variables; *M*, Mean; *SD*, standard deviation; *χ*^2^, value of the chi-square test; *t*, value of the *t*-distribution; *df*, degrees of freedom; *p*, probability value.

### Primary outcomes: Intervention effectiveness

#### Effect of intervention on affective attitude (Hypothesis 1a)

The 2 × 2 repeated measures ANOVA (see Table 2 for means and standard deviations by condition over time) showed neither a significant condition x time interaction (*F*(1,71) = 0.12, *p* = 0.727), nor any significant main effect [main effect of time: *F*(1,71) = 0.19, *p* = 0.661; main effect of group: *F*(1,71) = 0.18, *p* = 0.670]. Thus, Hypothesis 1a was not supported.

**Table 2:** Means and standard deviations by condition over time (*N* = 73).

	Initial assessment		Final assessment	
	Affect	Control	Affect	Control
SRHI	3.41 (0.84)	3.47 (0.78)	3.56 (0.73)	3.65 (0.68)
SRBAI	3.33 (1.08)	3.26 (1.06)	3.54 (1.01)	3.64 (0.70)
Affective attitude	5.70 (0.99)	5.82 (0.10)	5.79 (0.98)	5.83 (0.88)

Habit strength was measured on a 5-point Likert scale; affective attitude on a 7-point scale.

#### Effect of intervention on habit strength (Hypothesis 1b)

The 2 × 2 repeated measures ANOVA showed no significant condition x time interaction [*F*(1,71) = 0.05, *p* = 0.825]. The only significant main effect was the main effect of time [*F*(1,71) = 5.20, *p* = 0.026, *η^2^* = 0.07]. This means that independent of condition, exercise instigation habit strength as measured with the SRHI significantly increased over time (Table 2). The main effect of group was not significant [*F*(1,71) = 0.22, *p* = 0.641]. Also when analyzing the SRBAI-scores, the only significant effect was the main effect of time [*F*(1,71) = 6.91, *p* = 0.011, *η^2^* = 0.09]. In sum, Hypothesis 1b was not supported.

#### Effect of intervention on affective response to exercise (valence) and automaticity (Hypotheses 1c, d)

The means of the weekly measured variables included in the proposed models are presented in Table 3. Throughout all of the weeks, mean affective valence in the affect condition was 7.92 (*SD* = 1.04, range 5–10) and 7.64 (*SD* = 1.15, range 4–10) in the control condition; mean automaticity in the affect condition was 7.06 (*SD* = 2.27, range 1–10) and 7.08 (*SD* = 2.19, range 1–10) in the control condition.

**Table 3:** Means of weekly measured variables.

Week	1	2	3	4	5	6	7	8	9	10
*n*	117	97	95	85	81	83	82	51	65	61
Valence	8.04	7.84	7.55	7.76	7.53	7.65	7.62	7.63	7.45	7.95
Automaticity	6.67	7.61	7.26	7.18	7.32	7.58	7.87	7.71	7.54	7.39

*N* = 132 in week 1, n, valid cases. Valence and Automaticity were measured on a 10-point scale.

**Figure 1:**
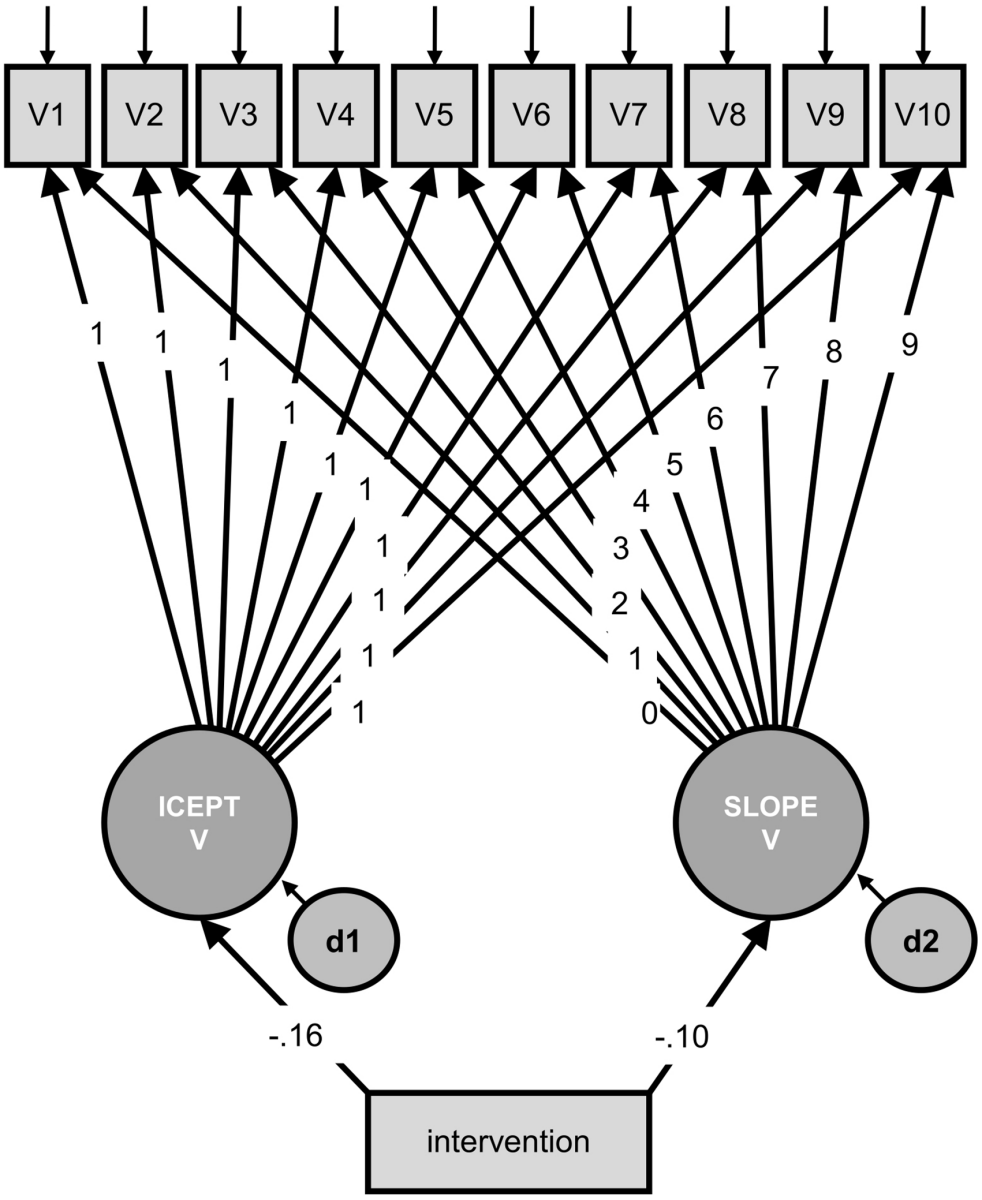
Model for valence with intervention as predictor variable. V1–V10 = observed valence, measured at 10 time points, with residuals; ICEPT V = latent intercept of valence, all paths to the observed valence variables were constrained to 1; SLOPE V = latent slope of valence, the paths to the observed valence variables indicate linear growth; d1 and d2 = residuals; intervention = observed predictor variable. Model fit: *χ^2^* = 65.22, *df* = 59, *p* = 0.269; CFI = 0.94; RMSEA = 0.03 (90% CI 0.00–0.06).

Regarding the model for valence with intervention as a predictor variable (Figure 1), chi-square statistics indicated that there was no significant difference between the postulated model and the data [*χ^2^* = 65.22, *df* = 59, *p* = 0.269; CFI = 0.94; RMSEA = 0.03 (90% CI 0.00–0.06)]. Given the intervention’s non-significant effect on the intercept factor of valence (*β* = −0.16, *z* = −1.16, *p* = 0.245), the intervention was not found to be a significant predictor of valence in week 1. Also, according to the intervention’s non-significant effect on the slope factor of valence (*β* = −0.10, *z* = −0.44, *p* = 0.660), the intervention was not found to be a significant predictor of the rate of change in valence. That is, neither the initial value nor the growth pattern of valence was related to the intervention. Hypothesis 1c was not supported.

**Figure 2:**
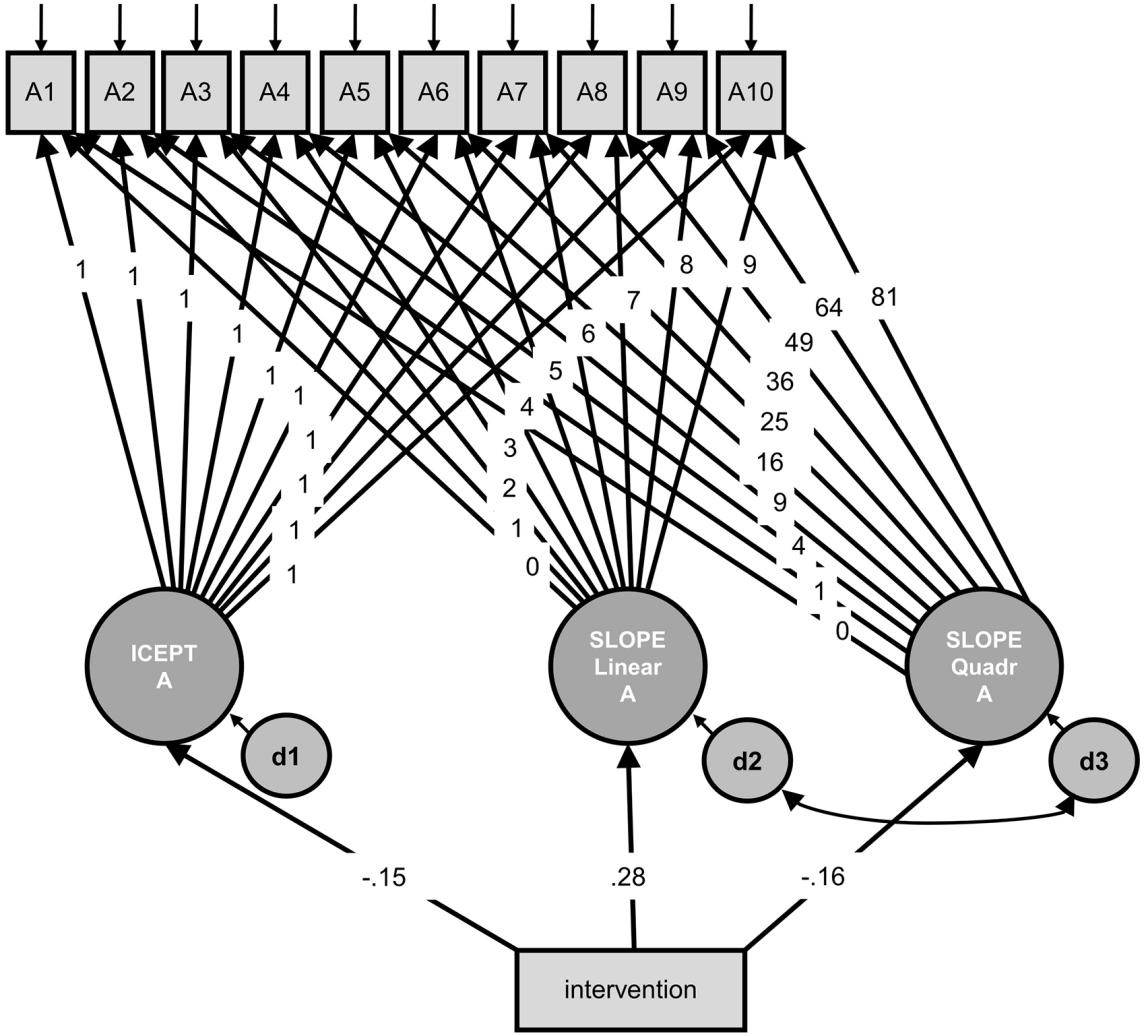
Model for automaticity with intervention as predictor variable. A1–A10 = observed automaticity, measured at 10 time points, with residuals; ICEPT A = latent intercept of automaticity, all paths to the observed automaticity variables were constrained to 1; SLOPE Linear A = latent slope of automaticity, the paths to the observed automaticity variables indicate linear growth; SLOPE Quadr A = latent slope of automaticity, the paths to the observed automaticity variables indicate quadratic growth; d1–d3 = residuals; intervention = observed predictor variable. The model revealed acceptable fit indices.

The model for automaticity (with a single headed path between the two slopes, Figure 2) with intervention as predictor variable revealed acceptable fit indices [CFI = 0.93, RMSEA = 0.06 (90% CI 0.03–0.09)] with *χ^2^* = 81.23, *df* = 55, *p* = 0.012. Again, all effects were not significant (intercept: *β* = −0.15, *z* = −1.12, *p* = 0.262; linear slope: *β* = 0.28, *z* = 1.61, *p* = 0.107; quadratic slope: *β* = −0.16, *z* = −0.96, *p* = 0.338), indicating that the intervention neither effected the initial level of automaticity nor its change over time. Hypothesis 1d was not supported.

### Secondary outcomes: Relationship between valence and habit strength (Hypothesis 2)

**Figure 3:**
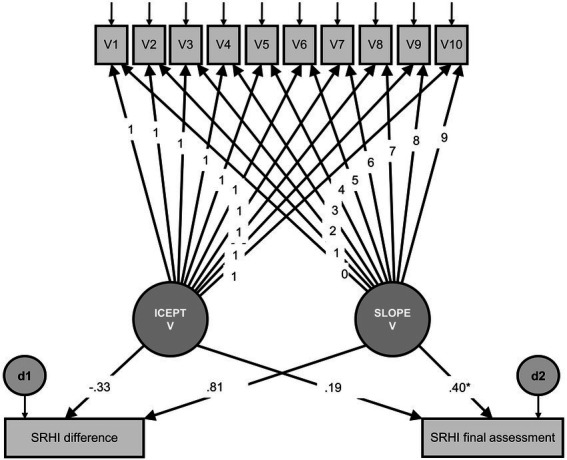
Model for valence as a predictor of final habit strength and change in habit strength. V1–V10 = observed valence, measured at 10 time points, with residuals; ICEPT V = latent intercept of valence, all paths to the observed valence variables were constrained to 1; SLOPE V = latent slope of valence, the paths to the observed valence variables indicate linear growth; d1 and d2 = residuals; SRHI difference = observed change in habit strength (final assessment minus initial assessment); SRHI final assessment = observed habit strength in the final assessment; *p < 0.05; **p < 0.01; ***p < 0.001. Model fit: *χ^2^* = 74.79, *df* = 68, *p* = 0.268; CFI = 0.94; RMSEA = 0.03 (90% CI 0.00–0.06).

Mean change of habit strength (SRHI) was 0.17 (*SD* = 0.62, range −1.25–2.50, *N* = 73). The model for Hypothesis 2 is shown in Figure 3.

Chi-square statistics indicated that there was no significant difference between the postulated model and the data [*χ^2^* = 74.79, *df* = 68, *p* = 0.268; CFI = 0.94; RMSEA = 0.03 (90% CI 0.00–0.06)]. The effect of the slope of valence on exercise instigation habit strength measured with the SRHI was significant (*β* = 0.40, *z* = 2.00, *p* = 0.045). This means that the rate of change in valence was a significant predictor of the final exercise instigation habit strength score. All other effects were not significant, indicating that the intercept of valence did not predict final exercise instigation habit strength (*β* = 0.19, *z* = 1.28, *p* = 0.201) and that neither slope (*β* = 0.81, *z* = 1.06, *p* = 0.288) nor intercept (*β* = −0.33, *z* = −1.94, *p* = 0.052) of valence predicted the difference score of exercise instigation habit strength. Also when analyzing the SRBAI-scores we found the effect of the slope of valence on exercise instigation automaticity strength to be significant (β = 0.50, *z* = 2.49, *p* = 0.013). Additionally, here, the intercept (*β* = −0.41, *z* = −2.37, *p* = 0.018) of valence predicted the difference score of exercise instigation automaticity strength. In sum, Hypothesis 2 was supported.

The non-significant path between the SRHI difference (final minus initial assessment) and the slope of valence should be interpreted with caution. One scenario in which gain scores are valid is where the post-test variance exceeds the pretest variance (May and Hittner, 1997). However, the inter-individual variance of the final SRHI score was lower than that of the initial SRHI score. There is a risk that in the initial assessment the participants related the items used in the SRHI to another course, resulting in a greater variance than in the final week, where it is reasonable that, when answering the SRHI, the participants referred to the current exercise course within the study period. The same is true for the SRBAI difference score.

## Discussion

The purpose of this study was, first, to examine whether a trainer-focused affect-based intervention could increase (a) affective attitudes, and (b) exercise instigation habit strength, and influence the development of (c) weekly measured affective responses, and (d) weekly measured automaticity among adult exercise course participants. Second, the study examined the relation between the development of affective responses to exercise and habit strength.

With regard to the first set of Hypotheses (1a–d), the intervention did not show any effect on the affective constructs affective attitude and affective response. This was contrary to what was expected. These hypotheses were mainly based on a study by Jekauc (2015), which successfully manipulated affect with a similar intervention. Regarding habit formation constructs automaticity (SRBAI) and habit strength (SRHI), the affect-based intervention was no more conducive for habit formation than the control intervention. Our assumption that an affect-based intervention would be more beneficial for habit formation than a control intervention was mainly based on a study by Weyland et al. (2020) that suggested a relationship between valence and automaticity, which led us to conclude that an affect-based intervention might also influence habit formation.

Regarding habit formation constructs, results of the present study showed that exercise instigation habit strength significantly increased over time – independent of condition and when measuring habit with both the SRHI and the SRBAI. That behavior and habit strength increased over three time points for all participants independent of whether they were in self-monitoring or cue-to-action conditions was shown in a study by Mergelsberg et al. (2021). In their study, they wanted to compare effects of different conditions on habit formation of a new health behavior, namely microwaving a sponge or dishcloth, and behavior implementation, but then concluded that all conditions were equally effective. That is, also their habit monitoring condition, in which participants answered the SRHI about the health behavior under study every three days for three weeks, developed a habit equally to the other conditions. In our study, the increase in exercise instigation habit strength might probably have occured due to the weekly self-monitoring of automaticity through the weekly questionnaires. Another possible reason for the increase in habit strength is that PA behavior is generally more likely to become habitual, considering, for example, the stability of the exercise course’s context. A meta-analysis already showed that the highest habit strength, around 60 percent above the SRHI mean, can be found in relation to PA behavior in comparison to dietary behavior (Gardner et al., 2011). Further, the increases in SRHI scores found in this study might also be explained by the behavior repetition itself. The SRHI applied in this study includes items on behavior frequency and, thus, it may be that while frequency has increased, automaticity may not have (but see also Gardner et al., 2012; and Rebar et al., 2018). Regarding automaticity, our study’s results differ and therefore it is unclear whether the increase in habit strength is attributable to automaticity: The main effect of time was also significant when analyzing the SRBAI-scores suggesting that exercise instigation automaticity strength significantly increased over time – and not only the frequency of behavior. Conversely, there was no significant growth over time in our study regarding the weekly measured automaticity, supporting the suggestion that the increase in habit strength reflects more of an increase in behavior frequency than in automaticity. That automaticity did not grow significantly is a finding that is contrary to other research that has reported, for example, asymptotic growth in automaticity (Lally et al., 2010). The courses being analyzed had all just started at the beginning of the semester. Still, it is possible that some courses were offered in a comparable setting the previous semester, which would be a possible explanation for the high values of automaticity already perceived in the initial assessment. On the other hand, given the different results for automaticity, there may have been methodological problems, such as low reliability and validity of automaticity self-reports. As Labrecque and Wood (2015) also noted, unlike the frequency of a behavior, an individual cannot directly observe automaticity. They conclude that automaticity self-reports can only capture inferences about the feeling of performing a behavior, which could misrepresent the underlying inherently unconscious characteristic of habits.

With respect to the affective constructs, the present study revealed no significant growth in valence over time and no inter-individual difference in the growth patterns regarding valence. Thus, a statistical explanation for the ineffectiveness of the intervention may lie in the lack of variance in valence, which can be attributed to two methodological aspects. First, the generally rather high means in valence might be attributed to the affective rebound. The affective rebound describes the consistent finding that people generally feel more positive valenced states after finishing exercise or when resting between exercise-intervals (Hall et al., 2002; Backhouse et al., 2007; Box et al., 2020). Measuring affective responses at several time points during and after exercise is therefore recommended (Ekkekakis et al., 2020). However, this is difficult to implement in real-life settings, such as structured university sport and exercise courses. Given the lack of variance between the persons due to the positive rebound, further gains in valence cannot be achieved easily. Second, a self-selected behavior, as in this study, may reduce inter-individual variance in the reward value, in this case positive affective responses, since individuals might have chosen exercise courses regarding which they anticipated positive affective responses. This argumentation can also be found in Schnauber-Stockmann and Naab (2019), who gave subjects a specific and not self-selected app in order to increase inter-individual variance in the app’s reward value to study it as a facilitator of habit formation.

Hypothesis 2 stated that the development of positive affective responses to exercise would be positively related to the development of habit strength. The results should be taken with caution in light of the low variance in valence, but they do suggest that subjects with an increase in valence also had a stronger exercise instigation habit strength reported in the final assessment. Positive affect might be associated with the formation of habits, as also assumed by Weyland et al. (2020) who found significant effects of valence on automaticity on the between-subject level. Wood and Neal (2016) summarize that uncertain rewards that do not occur every time are most effective for habit formation. Since one cannot be certain of a positive valence as an outcome of the exercise course, the affective response might represent an uncertain reward. Focusing on intrinsic rewards and also applying a latent growth model, one study found that the intercept of intrinsic rewards in the preparation phase (i.e., finding it pleasant) was associated with the intercept of exercise preparation habit strength, but not with its slope (Lee and Yoon, 2019). The authors conclude that rewards occurring especially early are related to habit formation, and the influence of rewards might then decrease during habit change. Also focusing on intrinsic exercise rewards (measured as intrinsic motivation and negative reinforcement), Phillips et al. (2016) underlined the importance of the relationship between habit and rewards for the actual behavior. In particular, they found that exercise habit strength mediated the relation between intrinsic exercise rewards and exercise behavior for individuals who had done regular exercise for at least three months. For individuals who exercised less than three months, however, this relation was mediated by intentions. However, given the different variables, these results are hard to compare and none of these studies assessed acute affective responses per se.

### Limitations and future research

A strength of the study is that it examined adults in a real-world setting over a ten-week period with respect to the emergent variables of affect-based constructs and habit, which are promising for behavior change (Conner et al., 2020; Orbell and Verplanken, 2020). Only a few studies actually measured habit strength or automaticity at multiple time points (Feil et al., 2021).

Another strength of this study is that it is an intervention study that intended to manipulate affective responses through exercise trainers. This trainer-focused approach might reach more individuals than interventions that need to be perceived by single individuals, for example exercise course participants. Authors like Ekkekakis and Zenko (2016) argue that exercise psychology should produce efficient implementable interventions for a wide range of settings, not least to increase the impact of the discipline. However, because the intervention did not yield significant results, it is necessary to list possible reasons why the intervention might have failed. We based the intervention on facilitators of positive affective responses to exercise (Wienke and Jekauc, 2016), but still it is possible that the intervention content was not sufficiently relevant for changes in affect. It is possible that the focus of such an intervention should have been task-oriented teaching styles (Klos et al., 2020) or other affect-based intervention techniques (Chen et al., 2021). In this context, it seems important for future studies to examine which techniques are most effective in manipulating affective constructs – and whether these techniques are also most beneficial for habit formation (see also question 13 of the 21 questions to guide future research by Gardner et al., 2021). Regarding study design, one limitation in this study was that two different interventions were compared, with one intended to serve as a control intervention. Since we speculate that the affect-based intervention might not have focused on the most relevant aspects, future studies should compare similar intervention components in order to gather insights into which contents are effective rather than concluding that an intervention as a whole is (in-) effective as inspired by Gardner et al. (2020a). Another explanation for the ineffectiveness of the intervention is that the initial workshop lasted only 1 h and thus may have been too short for any reflection by the exercise trainers on their own behavior.

At the same time, it can also be argued that the intervention did not fail, but that its effect could not be demonstrated for methodological reasons. First, we did not measure whether the exercise trainers designed their exercise courses as recommended by the affect-based intervention. Thus, given this lack of a “manipulation check,” it remains unclear whether the present results are due to an insufficient theoretical foundation of the intervention or to an insufficient implementation of the intervention by the exercise trainers. With the goal of verifying actual implementation, future studies could record some of the exercise courses and analyze the videos by asking experts to determine whether the intervention content was applied (see, for example, González-Cutre et al., 2018). Second, possibly, the effect of the intervention was too small to be detected statistically, given the small sample size in which confounding variables that were not controlled for may play a role in masking the intervention effect. One such determinant of affective responses could be, for example, BMI. Obesity can be associated with factors that may result in reduced enjoyment of exercise in obese individuals (Ekkekakis et al., 2017). Notably, the high dropout rate in itself can be interpreted as the absence of the theoretically expected consequences of the intervention, since positive affect was assumed to counteract it. Third, we can conclude that the intervention had no effect on affective valence measured weekly *after* exercising. It is recommended that future studies expand the measurement time points, i.e., measure affective responses *during* exercise. It is questionable to what extent an intervention, which focuses on situational factors that are to be influenced by an exercise trainer’s behavior during the course, influences post-exercise affective responses. Moreover, as far as the predictability of future PA is concerned, affective responses during exercise are shown to be more reliable (Rhodes and Kates, 2015).

Although we found an increase in affective valence and final exercise instigation habit strength to be significantly related, since the affect manipulation in this study was not successful, no conclusions about the direction of this relationship can be drawn. It is possible that positive affective responses enhance habit formation. However, it is also reasonable that the more automatic a behavior is instigated, the more positive the affective responses are that accompany this behavior. However, the latter argument is more likely to be found in relation to execution habit, which was not measured in the present study. Gardner et al. (2020b) hypothesize that execution habit might influence the uptake of future PA via positive affective judgments, among other mechanisms.

Another critical point, in addition to the discussion of the extent to which implicit processes can be recorded by self-report (see for example Gardner and Tang, 2014; Hagger et al., 2015), is the choice of the SRHI item stems. Only recently have recommendations for habit formation tracking studies been published that suggest measuring specific behaviors in light of the specific context in which they occur. For example, by including a potential cue in the item stem of the SRHI/SRBAI (Sniehotta and Presseau, 2012; Gardner et al., 2021; e.g., “Going to the gym after the lecture on Wednesday is something…”). In this study, we assumed that the overall contexts were stable, given that time and place were constant for all exercise courses. Nevertheless, we did not assess the individual cues a person relied on when instigating the behavior. Further, there was a short Christmas break within the study period (between week 7 and 8), which arguably affected behavior frequency and consistency within a given behavioral sequence.

### Conclusion

In conclusion, the present study assessed the importance of affective responses in the formation of instigation habits in exercise contexts and discusses possible mechanisms for affect-based interventions. Although the trainer-focused intervention was not successful in increasing positive affective responses in course participants, we found a significant relationship between the development of weekly affective responses and habit strength at the end of the intervention. We encourage future studies to follow this line of research. In particular, in line with current dual-process approaches, investigating the nature of the relationship between affect and habit might contribute to a better understanding of the processes related to PA maintenance.

## Data availability statement

The raw data supporting the conclusions of this article will be made available by the authors, without undue reservation.

## Ethics statement

The studies involving human participants were reviewed and approved by Karlsruhe Institute of Technology Ethics Committee. The patients/participants provided their written informed consent to participate in this study.

## Author contributions

SW: conceptualization, data curation, formal analysis, investigation, methodology, project administration, resources, visualization, writing – original draft preparation, and writing – review and editing. JF: conceptualization, formal analysis, and writing – review and editing. KF: conceptualization and writing – review and editing. DJ: conceptualization, formal analysis, methodology, supervision, and writing – review and editing. All authors contributed to the article and approved the submitted version.

## Funding

We acknowledge the support by the KIT-Publication Fund of the Karlsruhe Institute of Technology.

## Conflict of interest

The authors declare that the research was conducted in the absence of any commercial or financial relationships that could be construed as a potential conflict of interest.

## Publisher’s note

All claims expressed in this article are solely those of the authors and do not necessarily represent those of their affiliated organizations, or those of the publisher, the editors and the reviewers. Any product that may be evaluated in this article, or claim that may be made by its manufacturer, is not guaranteed or endorsed by the publisher.
