# Supplementary figures and images for: Investigating the relation between positive affective responses and exercise instigation habits in an affect-based intervention for exercise trainers: A longitudinal field study
Source: Front Psychol. 2022 Sep 23;13:994177. doi: 10.3389/fpsyg.2022.994177 (PMC9540191; doi:10.3389/fpsyg.2022.994177)

## Slide 1
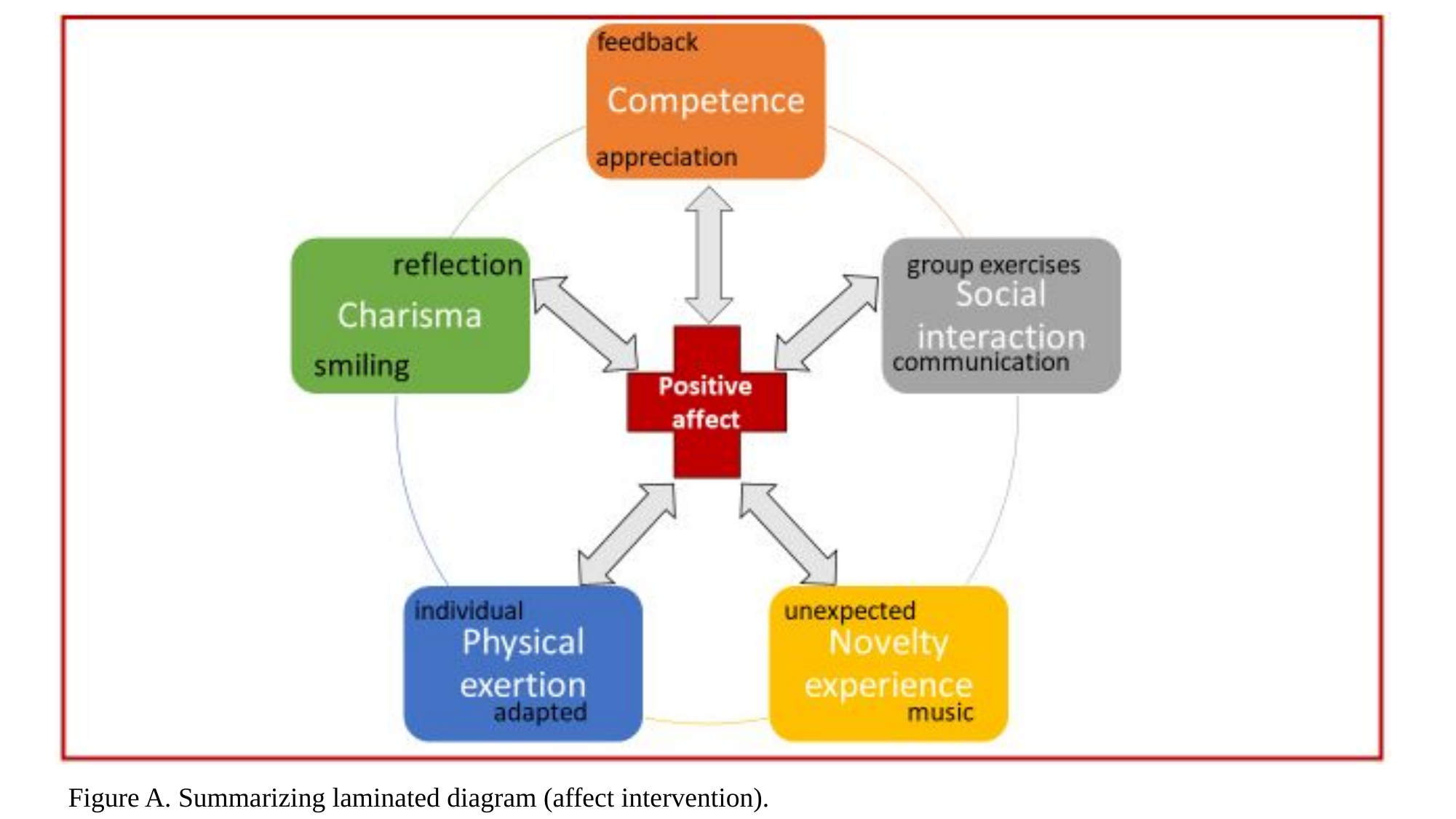

Figure A. Summarizing laminated diagram (affect intervention).

Supplement: Supplementary file 1 [file Presentation_1.pptx]
