# Supplementary material for: Investigating the relation between positive affective responses and exercise instigation habits in an affect-based intervention for exercise trainers: A longitudinal field study
Source: Front Psychol. 2022 Sep 23;13:994177. doi: 10.3389/fpsyg.2022.994177 (PMC9540191; doi:10.3389/fpsyg.2022.994177)
Supplement: Supplementary file 2 [file Presentation_2.pptx]

## Slide 1
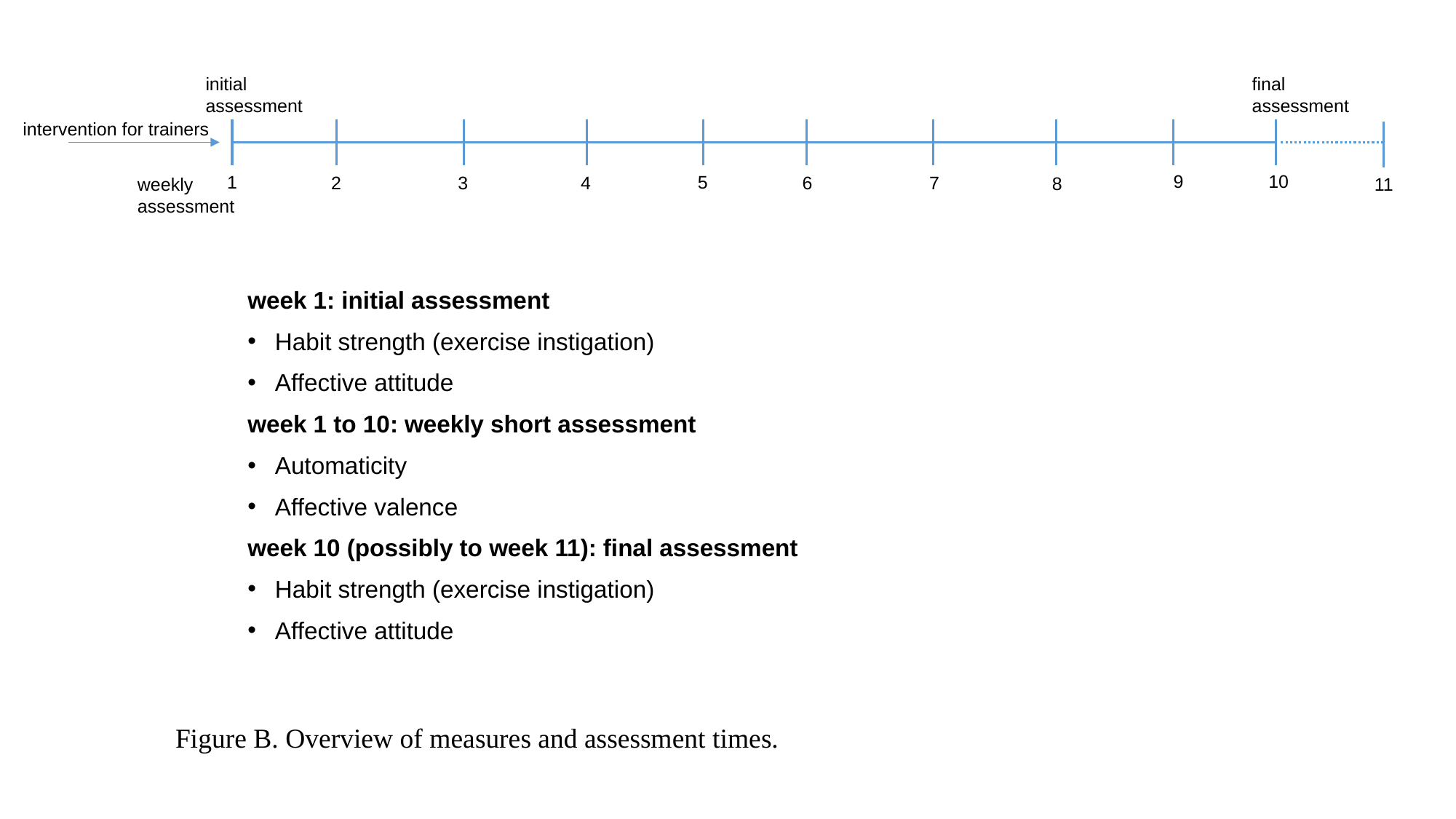

initial
assessment
final
assessment
10
9
5
1
2
3
4
6
7
8
weekly assessment
intervention for trainers
11
week 1: initial assessment
Habit strength (exercise instigation)
Affective attitude
week 1 to 10: weekly short assessment
Automaticity
Affective valence
week 10 (possibly to week 11): final assessment
Habit strength (exercise instigation)
Affective attitude
Figure B. Overview of measures and assessment times.
