# Supplementary material for: Investigating the relation between positive affective responses and exercise instigation habits in an affect-based intervention for exercise trainers: A longitudinal field study
Source: Front Psychol. 2022 Sep 23;13:994177. doi: 10.3389/fpsyg.2022.994177 (PMC9540191; doi:10.3389/fpsyg.2022.994177)
Supplement: Supplementary file 3 [file Table_1.docx]

**Appendix A**

**How sport can be even more fun**

Physical activity is good for your health. So far, so good. That's no big surprise and most people are aware of it. Therefore, you should exercise more often, be more physically active in your everyday life, and sit less, especially after work. In addition, you should eat plenty of fruit, avoid sugar and meat, drink plenty of water, and go outside more often to breathe fresh air. So many things to keep in mind. However, a Friday evening on the sofa with beer and crisps is a lot of fun. The gym smelling of sweat just can't keep up, and there is always tomorrow. So many people intend to replace their unhealthy habits with good ones and want to lead a healthier life, but then fail to transfer these intentions into behavioral changes. And who can blame them? Who has never personally experienced this before? After all, it's not that simple. If we assume that we strive to have a good time and to get the most out of life, then it's obvious that we prefer the sofa to sport, as being physically active is not quite as much fun.

But sport does not have to lose this competition with the sofa! Exercising or doing sport should be beneficial to health, but above all it should be fun. After all, if it is accompanied by enjoyment and pleasure, people are more likely to repeatedly engage in physical activity. Who wants to sit at home when it is so much more fun to give it your all together with nice people, to go all out, but also to get to know your own limit, to experience new activities, and to enjoy the good feeling of being in the shower after a sense of achievement in training?

Well, good, so exercising is supposed to be fun, sure. It’s not like you as a trainer did not already know that. And of course it’s not exactly your intention to stand on the sidelines as grumpily as possible and maltreating the course’s participants. We don't assume this of you, either. But even if you've already created an appealing course program, there are certainly still a few simple adjustments that can be made to make it even more fun. Sometimes, you may simply lack the time to prepare the ideal program. Sometimes, there might be a lack of new ideas, and sometimes, you might just have come home from a stressful day and thus have no energy left to spread joy in your course. We take all this into account and want to give a few simple, feasible impulses that will not only make the course more fun for the athletes, but also more fun for you as a trainer. Because that is not to be neglected: you should also enjoy everything.

Five factors are described below. The behavior of each of these categories can increase the level of fun. However, it is not necessary to implement all the behaviors described in every single course session. You may decide to try one behavior one week and another the next. Some of the ideas mentioned may not appeal to you personally, because they just don't fit you as an individual. That's okay, no one wants to completely change you and force behaviors onto you that alienate you. From everything listed below, pick what you feel like doing, what makes sense to you, and what you might have wanted to try anyway.


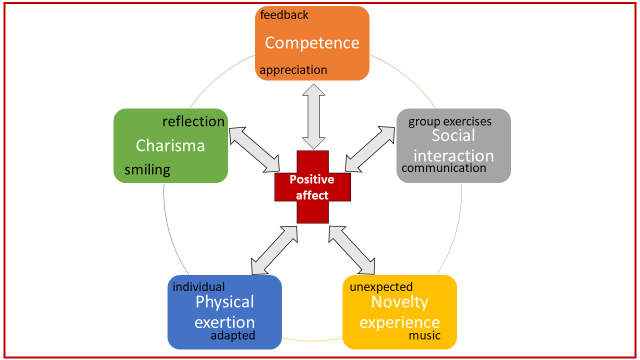


**Competence**

**
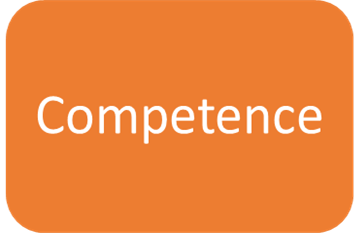
** **Provide positive feedback**

feedback

**Set realistic goals (individually and in the group)**

**Appreciation**

**Adapted challenges**

appreciation

**Competition**

**Praise development**

This is not about your competence as a coach, we don't doubt that at all. This is about the perceived competence of the athletes. In other words, it's about them having the impression that they're really good at what they do, that they're really successful at sports. Winning is fun, being good is fun. And when we are endorsed in what we do, we like to do it again.

In order for the athlete to know exactly, and without doubting herself, that she is good, she needs her coach’s feedback. Generally speaking, emphasizing the positive makes for a better mood than always focusing on what is not yet working so well. It helps to divide a movement into small steps. Even if three out of four individual steps are not yet perfect, it is worthwhile to positively emphasize the part that is already working really well. If the ultimate goal is a long throw or a perfect jump, this can only be achieved when everything perfectly fits into place. That would be at the very end of an intensive training period. The worst case would be if the athlete never reaches this ultimate goal. Along the way, it is important to aim for small, more realistic goals and to give praise when these milestones have been reached. Especially when the individual is not in their best form yet, it can help to focus on the group and praise its progress as a whole.

In addition to providing positive feedback, appreciation is an important factor. This involves, for example, paying close attention to how the athlete behaves in training, what improvements have been made and how intensively she trains. Even if something doesn't work out, you can appreciate the commitment and perseverance. Again, communicate! The athlete will certainly be happy to hear that her commitment in training has been noticed and appreciated. A guiding principle here can be, "Don't criticize, accept." This means that one must also sometimes accept that the targeted performance has not been achieved, without immediately highlighting this in a negative way. Of course, the point is not to gloss over everything. But even stagnation or setbacks can be communicated in an appreciative rather than critical manner. Appreciation is also conveyed by respecting other opinions and showing interest in them, by listening persistently. This conveys to the athletes that, with their opinions, they can contribute something to the training, that they are therefore worth something and give off a sense of competence. For example, when faced with a new challenge, athletes can be asked for their ideas on how to solve it, rather than providing the answer as a coach.

When it comes to providing positive feedback and appreciating athletes, it is of course also important to enable progress in training in the first place so that it can then be praised. No one should be under or over challenged. The skill is to ideally dose the challenges for each individual. One person tries to complete the exercise in five minutes, the other in six. The competition with time or other small competitions that are built into the training (e.g., achieving a certain number of repetitions) spurs them on and makes progress visible. Through such specific competitions, you can quite clearly see when someone has improved. These constant, small individual improvements are a success that should be rewarded!

**Social interaction**

**
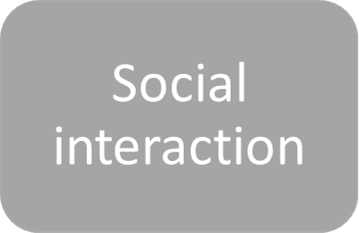
** **Group/partner exercises**

group exercises

**Interdependence**

**Encourage communication**

**Push each other**

**Rituals**

communication

**Social Events**

Sure, some people simply enjoy being alone whilst going out for a walk. It's quiet, they don't have to talk to anyone and they can switch off. But the moment the participants in your course decide to go to an exercise course in which others are also taking part, it is obvious that they will not be alone. The opposite becomes true: being alone within a group is frustrating. When the others form a team and you yourself have the impression that you don't really belong, it's hard. After all, it's really fun to form a team. Together, individual exercises may be more successful, the good mood of the others pushes the individual. *Me* turns into *us*. In addition, it's harder to quit the course when the others are counting on you.

One example of course elements that strengthen the sense of togetherness is group exercises. This is obvious in team sports. You can also do exercises in pairs that are actually individual. To avoid the unpleasant feeling of someone not finding a partner, as a trainer, you can also divide up the teams yourself. There are two possibilities with group exercises: several athletes try to reach a goal together or they compete with each other. Both can promote fun. In the case of group competitions, however, the losing team should also be appreciated and under no circumstances should it be humiliated. It is also important to mix up the teams from time to time so that two competing subgroups do not form within one course group.

Here, too, the key is communication at eye level. Conflicts in the course should always be addressed and discussed immediately. Again, it is about appreciation and trust. That's why it's important to promote communication, to encourage participants, and to address things.

However, it is not only the coach who has the task of providing positive feedback. Athletes can also praise each other. They may be uncomfortable with this at first, or they may simply not think of it, because they may not be used to praising others. Therefore, right at the beginning, the coach should encourage the athletes to push each other. This can include clapping or singing, because singing together creates a sense of community. Here again, it is important that no one is ever laughed at!

Moreover, rituals can contribute to a sense of togetherness. For example, greeting each other or bidding each other farewell can always be done with the same words, to which the group forms a circle. Also, cheering can be done through a battle cry which is the same every time.

Of course, social activities outside of the course can also bring the group together. If it seems appropriate and the group is ready to meet outside of the sports facility, such activities can be supported by the trainer. The important thing is that no one is excluded. If someone didn't want to or couldn't attend the social event, he or she should still be considered an equal team member during training. This can also be addressed by the coach to take the pressure out of having to participate in social events.

**Novelty experience**

**
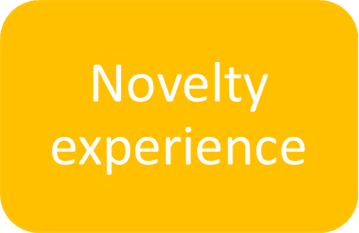
Music**

unexpected

**Unexpected**

**New accents**

**Medium level of variety**

**Nature experiences**

music

**Mindfulness**

By novelty experience we mean tackling boredom, meaning the course remains exciting, as every now and then something happens that no one expects. This creates excitement and curiosity that makes the participants want to come back next time. And, no wonder, it is of course more fun to experience something new more often.

In concrete terms, this can, for example, involve music or either funny or motivating videos shown during the warm-up. It is also a possibility to go outside into nature for a sport that usually takes place indoors. For a sport that takes place outdoors anyway, you could at least try new ways. Also, innovative exercises can make the course more diverse. Therefore, it can be tactically wise to not do all the different exercises in the first few course sessions, but to keep a few fun ones up your sleeve to show off once in a while. A good example for an unexpected novel experience are mindfulness practices. It doesn't have to be a meditative journey through the body if the group doesn't seem receptive to it. More "atypical" exercises are also possible, such as trying to line up a boiled egg or trying to find a walnut that someone had chosen from a bowl full of walnuts after mixing them all up. Another idea to add variety is to have one of the participants design and lead a warm-up exercise. It is surprising what exercise he or she comes up with and also the coach shows that he or she trusts the participant to have a certain competence. Anything that is unexpected and seems appropriate can provide variety!

It is important that despite the effort to provide variety, a certain routine is also created. It is therefore important to find a balance between habits and the unexpected.

**Physical exertion**

**
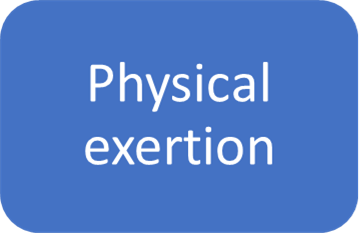
Medium intensity**

individual

**Individually adapted to fitness level**

**Observe**

adapted

Really testing yourself and pushing yourself to your limits can be fun. That feeling of falling into bed afterwards, completely exhausted, is priceless. But everything should be done with moderation and purpose. Of course, no one should collapse and be so exhausted that they think to themselves, "thanks, never again”. Overstraining is bad, but so is under-straining. If the intensity of the training is just right for the individual, the experience of competence increases and it is simply more fun. When the body is neither too exhausted nor not challenged at all, a specific feeling sets in which the athletes would like to experience again. To achieve this, it is helpful to observe each athlete closely and determine his or her individual fitness level. Who needs which time for which exercise? Who can repeat which exercise and how often? Whenever possible, the intensity of the training should be tailored to their fitness level.

**Charisma**

**
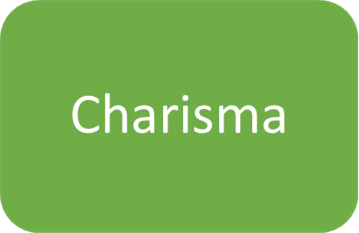
Positive emotions**

reflection

**Radiate enthusiasm**

**Reflection before the training**

**1 minute time**

smiling

Emotions are contagious! And that's not a bad thing at all; on the contrary, when one person laughs, the other joins in and suddenly no one can contain themselves. Enthusiasm is also transmitted very quickly. If the training is supposed to be fun, it is helpful for the trainer to also radiate fun, because if someone conveys positivity, the others want to be trained by him or her again and again. And yes, that's not always easy. It's also not about pretending or laughing artificially.

What can help to radiate positivity is to take a minute before each training. This prevents you from rushing straight from your own everyday life into the training session and thus transferring this stress to the others. In this minute, you can briefly reflect on your current state and how you feel. Once you have noticed your own affective state, it is important not to judge it, not to get angry when you are stressed. It is important to realize that the stress does not have to be a hindrance, you can still do very good training. To overcome your own affective state, it is important to know it. If you are receptive to good music, after the short reflection, you can listen to motivating music that gets you in the mood for training. As silly as it sounds: as a trainer, you have to get through the training, one way or the other, that is, whether with a smile on your face or a stress-distorted frown. And it is often the case that the positive affective state automatically comes with the smile - not least because the athletes reflect the smile of the trainer and the trainer then looks into smiling faces. And this creates a positive cycle!
