# Supplementary material for: Investigating the relation between positive affective responses and exercise instigation habits in an affect-based intervention for exercise trainers: A longitudinal field study
Source: Front Psychol. 2022 Sep 23;13:994177. doi: 10.3389/fpsyg.2022.994177 (PMC9540191; doi:10.3389/fpsyg.2022.994177)
Supplement: Supplementary file 4 [file Table_2.docx]

**Appendix B**

Descriptive analyses of missing values (item-nonresponses) revealed that for the 12 SRHI items for exercise instigation habit strength at week 1 (N = 132), 10 values (0.63 %) distributed among 9 subjects were missing; for the three affective attitude items at week 1, 3 values (0.76 %) distributed among 1 subject were missing. Among those participants who filled in the final questionnaire (N = 73), the analyses for the 12 SRHI items for exercise instigation habit strength revealed that 20 values (2.28 %) distributed among 7 subjects were missing; for the three affective attitude items, 3 values (1.37 %) distributed among 1 subject were missing. None of the Little’s MCAR tests, each calculated for all scales at both time points, was significant, suggesting that data were missing completely at random. It was therefore appropriate to use the expectation–maximization algorithm for data imputation to avoid list wise deletion in the case of analyses of variance (Dempster et al., 1977) and to use full-information maximum-likelihood estimation in the case of latent growth curve modeling (Arbuckle, 1996; Jekauc et al., 2012).

**References**

Arbuckle, J. L. (1996). Full information estimation in the presence of incomplete data. In G. A. Marcoulides & R. E. Schumacker (Eds.), *Advanced structural equation modeling: Issues and techniques* (pp. 243-277). Lawrence Erlbaum.

Dempster, A. P., Laird, N. M., & Rubin, D. B. (1977). Maximum likelihood from incomplete data via the EM algorithm. *Journal of the Royal Statistical Society: Series B (Methodological)*, *39*(1), 1-22. https://doi.org/10.1111/j.2517-6161.1977.tb01600.x

Jekauc, D., Völkle, M., Lämmle, L., & Woll, A. (2012). Fehlende Werte in sportwissenschaftlichen Untersuchungen. *Sportwissenschaft*, *42*(2), 126-136. https://doi.org/10.1007/s12662-012-0249-5
