# Supplementary material for: Investigating the relation between positive affective responses and exercise instigation habits in an affect-based intervention for exercise trainers: A longitudinal field study
Source: Front Psychol. 2022 Sep 23;13:994177. doi: 10.3389/fpsyg.2022.994177 (PMC9540191; doi:10.3389/fpsyg.2022.994177)
Supplement: Supplementary file 5 [file Table_3.docx]

|  | **1** | **2** | **3** | **4** | **5** | **6** | **7** | **8** | **9** | **10** | **11** | **12** | **13** |
| --- | --- | --- | --- | --- | --- | --- | --- | --- | --- | --- | --- | --- | --- |
| Sociodemographic information |  |  |  |  |  |  |  |  |  |  |  |  |  |
| 1. Gender | - |  |  |  |  |  |  |  |  |  |  |  |  |
| 2. Age | -.09 | - |  |  |  |  |  |  |  |  |  |  |  |
| 3. Student status | .08 | -.16 | - |  |  |  |  |  |  |  |  |  |  |
| Initial Assessment (week 1) |  |  |  |  |  |  |  |  |  |  |  |  |  |
| 4. SRHI week 1 | .17 | .02 | -.01 | - |  |  |  |  |  |  |  |  |  |
| 5. SRBAI week 1 | .18 | .05 | .03 | .89*** | - |  |  |  |  |  |  |  |  |
| 6. Affective attitude week 1 | .15 | -.13 | -.01 | .60*** | .61*** | - |  |  |  |  |  |  |  |
| Weekly questionnaire |  |  |  |  |  |  |  |  |  |  |  |  |  |
| 7. Mean valence | .04 | .03 | .05 | .32** | .37** | .35** | - |  |  |  |  |  |  |
| 8. Mean automaticity | -.10 | -.00 | .14 | .32** | .40*** | .31** | .42*** | - |  |  |  |  |  |
| Final assessment (week 10) |  |  |  |  |  |  |  |  |  |  |  |  |  |
| 9. SRHI week 10 | -.04 | -.02 | .11 | .67*** | .53*** | .56*** | .27* | .52*** | - |  |  |  |  |
| 10. SRHI difference | -.26* | -.05 | .14 | -.54*** | -.55*** | -.15 | -.11 | .17 | .27* | - |  |  |  |
| 11. SRBAI week 10 | -.08 | -.03 | .21 | .52*** | .53*** | .43*** | .29* | .73*** | .84*** | .28* | - |  |  |
| 12. SRBAI difference | -.27* | -.09 | .16 | -.52*** | -.63*** | -.28* | -.15 | .23 | .18 | .86*** | .33** | - |  |
| 13. Affective attitude week 10 | .00 | -.08 | -.04 | .46*** | .41*** | .54*** | .43*** | .41*** | .51*** | -.01 | .47*** | -.03 | - |
| *M (%)* | 17.8^a^ | 22.50 | 95.9 ^b^ | 3.44 | 3.30 | 5.76 | 7.81 | 7.53 | 3.60 | .17 | 3.60 | .29 | 5.81 |
| *SD* |  | 2.15 |  | .81 | 1.06 | .99 | 1.00 | 1.95 | .70 | .62 | .88 | .95 | .93 |

Table A. Means, standard deviations, and correlations among all included study variables (*N* = 73).

^a^percentage female. ^b^percentage yes. **p* < .05; ***p* < .01; ****p* < .001.
